# Monte Carlo simulation with confusion matrix paradigm – A sample of internal consistency indices

**DOI:** 10.3389/fpsyg.2023.1298534

**Published:** 2023-12-27

**Authors:** Yongtian Cheng, Pablo A. Pérez-Díaz, K. V. Petrides, Johnson Li

**Affiliations:** ^1^Division of Psychology and Language Sciences, University College London, London, United Kingdom; ^2^Instituto de Psicología, Universidad Austral de Chile, Puerto Montt, Chile; ^3^Department of Psychology, University of Manitoba, Winnipeg, MB, Canada

**Keywords:** Monte Carlo simulation, confusion matrix, null distribution, internal consistency, statistic comparison

## Abstract

Monte Carlo simulation is a common method of providing empirical evidence to verify statistics used in psychological studies. A representative set of conditions should be included in simulation studies. However, several recently published Monte Carlo simulation studies have not included the conditions of the null distribution of the statistic in their evaluations or comparisons of statistics and, therefore, have drawn incorrect conclusions. This present study proposes a design based on a common statistic evaluation procedure in psychology and machine learning, using a confusion matrix with four cells: true positive, true negative, false negative modified, and false positive modified. To illustrate this design, we employ an influential Monte Carlo simulation study by Trizano-Hermosilla and Alvarado (2016), which concluded that the Omega-indexed internal consistency should be preferred over other alternatives. Our results show that Omega can report an acceptable level of internal consistency (i.e., > 0.7) in a population with no relationship between every two items in some conditions, providing novel empirical evidence for comparing internal consistency indices.

## Introduction

Simulation studies use computer-generated data to investigate research questions (Beaujean, 2018). Monte Carlo simulation is a commonly used procedure that uses repeated random number selections to solve modeling problems (Gelfand and Smith, 1990). It is especially useful when a statistical assumption (e.g., normality) is violated or in situations without theoretical distribution (Fan, 2012). The Monte Carlo simulation was introduced to psychometrics by Patz and Junker (1999a,b).

Psychological researchers are often interested in determining the sampling distributions of test statistics, comparing parameter estimators (e.g., Cohen’s *d*), and comparing multiple statistics that perform the same function. In a Monte Carlo simulation context, a key factor is the design of the specific conditions to evaluate.

Different simulation studies use different designs with a variety of conditions. This is because the study’s aims usually dictate the selection of the conditions. Suppose a Monte Carlo study has been designed to test the violation of a certain assumption (e.g., normality). Both the condition that the assumption has been met (e.g., normal distributed population) and the condition that the assumption has been violated (e.g., skewed distributed population) should be included in the study. Now, assume a different study has been designed to test the performance of a statistic (or several statistics) across different population distributions. In this case, multiple population distributions should be included in the study design. In general, “the major factors that may potentially affect the outcome of interest should be included” (Fan, 2012, p. 437).

However, some recent studies overlooked the inclusion of the null distribution of statistics in the simulations. A null distribution of statistics represents a scenario with no estimated relationship between variables within a given sample (Hunter and May, 2003; Spurrier, 2003).

In this study, we advocate for including a null distribution of statistic conditions in the Monte Carlo simulation when evaluating and comparing statistical measures. Furthermore, we suggest that the performance of a statistic should be assessed in light of commonly used cut-offs. Psychologists often employ informal tests in their research to compare the statistics values to a pre-determined cut-off to reach a binary decision. For example, an area under the curve (AUC) greater than 0.7 in ROC analysis is considered the minimum acceptable threshold (Streiner and Cairney, 2007); A Root Mean Square Error of Approximation (RMSEA) of 0.08 is regarded as the upper limit for Structural Equation Model fitting (SEM, Fabrigar, 1999). An internal consistency (e.g., Cronbach Alpha) greater than 0.7 is considered an acceptable level of reliability, according to Taber (2018). Trizano-Hermosilla and Alvarado (2016) have conducted a Monte Carlo simulation study with a focus on internal consistency performance evaluation. In this paper, we will utilize the influential study by Trizano-Hermosilla and Alvarado (2016) as a practical example to demonstrate the inclusion of a null distribution and the assessment of the statistic using commonly used cut-offs.

This paper is organized into several sections. We review current practices regarding including null distribution in psychological Monte Carlo simulation studies and their associated limitations. Subsequently, we introduce a simulation design rooted in the confusion matrix as a proposed solution. The study conducted by Trizano-Hermosilla and Alvarado (2016) will be used as a practical example of this design. In conclusion, we engage in a comprehensive discussion about the design, supplemented by another illustrative sample.

## The null distributions conditions included in the Monte Carlo simulation psychological studies

We observed that the null distribution of statistics is generally included in existing Monte Carlo simulation studies in two ways: First, the null distribution of statistics is included to represent the condition that there is no true mean difference between two groups of scores and are usually referred to as conditions of null effect (e.g., Derrick et al., 2016; Carter et al., 2019; Fernández-Castilla et al., 2021). This is consistent with the suggestion of the American Psychological Association (APA) guidelines. That is, researchers should include the null distribution of statistics (i.e., no mean difference between two groups; Fan, 2012) in any simulation of effect to test and evaluate the potential threat of Type I error.

Second, the researchers include the condition of a null distribution in factors in the simulation (e.g., Heggestad et al., 2015). In Greene et al.’s (2019) study evaluating the bias of different kinds of fit indices, the authors manipulated (a) the strength of the cross-loadings between factors as 0, 0.1, 0.3, and 0.5, (b) the strength of the between-factor correlated residuals as 0, 0.1, 0.3, and 0.5, and (c) the strength of the within-factor correlated residuals as 0, 0.1, 0.3, and 0.5 in a model. In this sample, 0 represents the condition in which the relationship of cross-loadings or correlated residuals does not exist in the population of variables.

In summary, researchers commonly include the null distribution of the statistic condition when estimating a statistic’s performance closely related to the mean difference. For example, when examining Cohen’s d in a Monte Carlo simulation study, researchers typically include a condition of no mean difference between two populations. Researchers also include the conditions of null distribution in factors in simulation studies for statistical comparison. However, psychological researchers may sometimes neglect to include the null distribution of the statistic in some other circumstances, such as in cases where the examined statistic does not have a close relationship with the mean difference.

Returning to the fit indices study (Greene et al., 2019) one paragraph above, the authors should include conditions that a null distribution in factors, such as no between-factor correlated residual, and the conditions with the null distribution of the statistic, such that some simulated samples should have no relationship with the proposed model (i.e., no model fitting). In our view, the failure to include conditions of null distribution weakens the conclusion of the simulation in the study. This may occur because some researchers have not considered the performance of the statistic in the condition that the dataset follows a null distribution of this statistic. (i.e., how will the fitting index perform on random data?), although other researchers recognize its importance. For instance, Stone (2000, p. 64) points out: “In order to test statistically the fit of an item, it is then necessary to compare the statistic that is calculated with a null distribution.” Stone conducted a Monte Carlo simulation based on null distribution to compare goodness-of-fit test statistics in item response theory (IRT) models, and the results showed the superiority of the statistic he proposed. Fan and Sivo (2007) and Fisk et al. (2023) examined the performance of fit indices in structural equation modeling (SEM) under conditions of model misspecification. This misspecification refers to discrepancies between the theoretical structure of the model and the simulated dataset.

In summary, the null distribution of the statistic is widely included in NHST-related statistics. Yet, when evaluating a statistic that does not have a close relationship in NHST (e.g., fit indices), psychological researchers sometimes neglect the null distribution condition. This study demonstrates the importance of this issue using the example of an influential simulation study about the several common statistics of internal consistencies and will propose a new design based on a confusion matrix that always includes a test with null distribution in statistics and evaluates the statistics from these conditions. As our example, we have selected a study conducted by Trizano-Hermosilla and Alvarado (2016), which we will henceforth refer to as the “original study” for convenience.

## How will the missing null distribution of statistic conditions influence the result of a simulation study?

In the following section, we will offer a general overview of the original study. We will specifically address the shortcomings of not including null distribution of the statistics conditions in their simulation design and propose enhancements through the methodology developed in this study.

In the original study, Trizano-Hermosilla and Alvarado (2016) compared the performances of four internal consistency statistics: Cronbach’s Alpha, Omega (McDonald, 1999), GLB (Greatest Lower Bound, Sijtsma, 2009), and GLBa (Greatest Lower Bound algebraic, Moltner and Revelle, 2015). They made a comparison of these statistics with various normal and nonnormal distributions and two kinds of inter-correlation between items: tau-equivalent and congeneric.

The original study used Root mean square error (RMSE) and %bias as their criteria.


(1) $$\mathrm{RMSE}=\sqrt{{\frac{\sum \left(\hat{p}-p\right)}{Nr}}^{2}}$$

(2) $$\%\mathrm{bias}=\frac{\sum \left(\hat{p}-p\right)}{Nr}\times 100\%$$

where *p̂* refers to the observed statistics for each replication, *p* refers to the true value of statistics in the simulation population, and *Nr* refers to the number of replications. Larger absolute values in the RMSE and the %bias statistics indicate worse performance.

Based on the RMSE and the %bias, the authors reported that Omega showed the best performance across most conditions included in their study. In other words, when comparing the difference between observed sample statistics and the associated true population parameter values, Omega showed the smallest discrepancies across most of the conditions. This led the authors to conclude that Omega should be recommended as the preferred index of internal consistency in psychological research. Specifically, the original study suggests that Omega should be preferred over Cronbach’s Alpha, which is the most widely used measure of internal consistency. Various studies across multiple disciplines shared the opinion with the original study that Omega rather than Alpha should be used as an internal consistency measurement method (Watkins, 2017; McNeish, 2018; Cortina et al., 2020).

Importantly, for our purposes, Trizano-Hermosilla and Alvarado (2016) original study included only simulation conditions in which there was an effect measured by the statistic (i.e., populations with internal consistency). Specifically, it only included conditions with an acceptable level of internal consistency between items in the questionnaires (i.e., a true internal consistency of 0.731 and 0.845) for the condition of 6 and 12 questionnaire lengths, respectively. As mentioned above, Alpha and Omega values of 0.7 or above are indicated as acceptable internal consistency in psychological research (Taber, 2018). Therefore, it included the null effect of some factors (e.g., no distribution error). However, it did not include a null distribution statistic condition, as we suggest here. According to Tang et al. (2014), internal consistency refers to the degree of inter-item correlations among items with factor saturation. Thus, to simulate a null distribution for these internal consistency statistics, one can independently assign random numbers to each item.

As a result, we would argue that the conditions included in the original study are insufficient to support their conclusions. To elaborate, we propose a new hypothetical index, C, which is used to measure internal consistency, with 0.7 being set as an acceptable cut-off. C is a constant number that can be computed and observed across all the 1,000 simulated datasets. Suppose *C* is found to be 0.78 from each replicated sample, i.e., (3)


(3) C=0.78.

In other words, *C* is a dummy index without validity according to internal consistency estimation. However, based on the criteria employed in previous studies (i.e., RMSE and %bias), *C* has a similar level of error as the Omega index. Across conditions in the original study for length = 6 items, the population parameter of internal consistency is 0.731. This is based on Equations (1) and (2), in which *p* is always 0.731, and *p̂* is always 0.78. As a result, the RMSE is 0.049 (4)


(4) $$\text{RMSE}=\sqrt{\frac{\sum_{N_{r}=1}^{1,000}{\left(0.731-0.78\right)}^{2}}{1000}}=0.049$$

and the %bias is −4.9% (5)


(5) $$\%\text{bias}=\frac{\sum_{N_{r}=1}^{1,000}\left(0.731-0.78\right)}{1000}\times 100\%=-4.9\%$$

in all conditions. Across conditions included in the original study length of 12 items, with similar calculations, RMSE is 0.065, and the %bias is 6.5%. These two results will remain consistent regardless of other factors, like the type of distribution. Therefore, it appears that in a number of conditions, this dummy index can provide similar or even superior performance to the genuine indices included in the original study. Importantly, this indicates that based only on the empirical evidence provided in the original study, we cannot distinguish Omega from this dummy index *C*. *C* is an extreme theoretical case, and a statistic with a consistent number cannot be applied. However, A dummy index similar to C with variations can be simulated easily. For example, $\dot{C}$ can be simulated from a continuous uniform distribution [0.711, 0.751] and $\dot{C}$ also cannot be distinguished from Omega with the simulation conditions and criteria used in the original study.

To sum up, simulation studies often evaluate the performance of a statistic based on RMSE and %bias in Monte Carlo simulations, with a view to quantifying the distance between the sample estimates of an observed statistic and the true parameter values (i.e., TP) in the population. We agree that this approach can offer insights regarding the degree to which observed sample estimates are different from true population values. However, without the introduction of the null distribution of statistic conditions in simulation, researchers may reach incorrect or incomplete conclusions, as in the above example with the dummy *C* index.

To address this problem, we introduce in this study a Monte Carlo design based on criteria commonly used in psychology and machine learning to evaluate models with categorical or binary results: the “confusion matrix” (Marom et al., 2010). In psychology, researchers typically use a confusion matrix to evaluate the performance of a categorization model in real psychological practice (i.e., Ruuska et al., 2018). A confusion matrix comprises four quadrants: True Positive (TP), False Positive (FP), True Negative (TN), and False Negative (FN). Their relationships are shown in Table 1.

**Table 1:** The elements of a confusion matrix.

	Predicted true result	Predicted false result
Actual true result	TP	FN
Actual false result	FP	TN

True Positive (TP) is the proportion of results that correctly indicates the presence of a condition or characteristic in the population; True Negative (TN) is the proportion of results that correctly indicates the absence of a condition or characteristic in the population; False Positive (FP) is the proportion of results that erroneously indicates that a particular condition or attribute is present in the population, while False Negative (FN) is the proportion of results which erroneously indicates that a particular condition or attribute is absent in the population. In an ideal perfect model, TP and TN should be at 100%, and FN and FP should be at 0%.

We use the original study as an example to illustrate how to apply confusion matrix methodology to simulation studies in psychology, aiming at statistical comparison. The null distribution of the statistic in the original study is the condition where there is zero internal consistency between items in the population.

We also include the interpretation of results that come from the design. Internal consistency is both continuous and binary (i.e., cut-off), exemplifying the problem adequately. The present study will keep the original study’s design unless alternative designs better target the research problem, or the original design is not applicable.

### Simulation Study 1: estimating the true negative

As noted above, the original study does not include the null distribution of the internal consistency statistics. The original study only provides empirical evidence of TP. The following simulation aims to distinguish Omega from a dummy index like *C*. As suggested by several studies (Moriasi et al., 2007; Wang and Lu, 2018), for the continuous variable, RMSE and %bias can provide more information than a percentage. Therefore, we propose TN can also use these two criteria. In the case that Omega is an efficacy statistic or index and that TN conditions should have an RMSE close to 0 and a %bias close to 0%, upon which a dummy statistic or index like *C* should have an RMSE close to 0.70 and a %bias close to 70% in the TN condition. Therefore, using TN to test whether a statistic or index is merely a “dummy” one is crucial, and its inclusion in the simulation represents an important step toward obtaining truly conclusive results.

### Design

In the original study, the researchers simulated several factors, including sample size (250, 500, and 1,000) and item length (6 or 12). This study will also use the same design for these two factors. The original study also included the distribution of errors following Headrick’s (2002) that were introduced to 2, 4, or all 6 items of the 6-item condition and to 2, 4, 6, 8, 10, or all 12 items of the 12-item condition. However, Headrick’s method (2002) was not introduced to our current study to ensure there is no internal consistency created from this method between items and results and also for simplicity.

The original study included the tau-equivalent and congeneric models as simulation conditions. This aspect of the study does not apply to the condition of null distribution to internal consistency statistics. This is because there is no correlation between any two items in the null distribution population, regardless of its type. Therefore, this design is not included in our study. In summary, 2*3 = 6 conditions are included in the first simulation.

The original study simulated all datasets in R (R Core Team, 2021) with R Studio (Racine, 2012). The present study will also use these platforms (for details, please see Appendix). For each condition, the design of the original study was replicated 10,000 times. The current study will use the same replication time with 10,000 across six conditions.

Four kinds of internal consistency measurement indices were included in the original study: Alpha, Omega, GLB, and GLBa. As provided by the sample code in the original study, these functions were used in the original study to obtain the following results: omega$alpha for Alpha, omega$omega.tot for Omega, glb.fa$glb for GLB, and glb.algebraic$glb for GLBa. In addition, two packages were used in calculations in the original studies: Psych (Revelle, 2015) and GPArotation (Bernaards and Jennrich, 2015), which are also used in this study. Further, the Omega.total was used as the chosen index from Omega in the original study because Trizano-Hermosilla and Alvarado also reported and evaluated the performance of $\omega_{t}$ , and consequently, the present study will also make use of the same reliability index.

To create a null distribution of internal consistency, we simulated the dataset from a standard normal distribution N(0,1) for each item across participants during the replications. Accordingly, each item and each participant’s response are totally independent, which ensures that the true covariances and factor loadings in the population are always zero. To check the validity of this design, we followed Fan (2012, p. 436), who suggests, “We may do a quick data generation verification by generating a large sample.” We simulated a large dataset from N(0,1) and calculated four internal consistency indices, as they yielded results close to zero, which supported the simulated null distribution of statistics as accurate. This part of the code is provided separately. This study will also use RMSE and %bias as criteria, similar to the original study, to evaluate the performance of the statistics.

## Results

Our results indicated that none of these indices performed as a dummy index. However, according to the criteria used in the original study, Omega (i.e., Omega.total) performed the worst in some TN conditions and never the best. In contrast, Alpha showed the best performance across all conditions. This is possibly because Omega, by definition, cannot be smaller than zero, implying that errors can only inflate its results. The full results of our simulation are displayed in Table 2.

**Table 2:** Estimation of true negative in Study 1.

		%bias				RMSE			
Length	SS	Alpha	Omega	GLB	GLBa	Alpha	Omega	GLB	GLBa
6	250	12.41%	38.39%	15.89%	30.67%	14.17%	39.35%	17.58%	32.53%
6	500	9.00%	33.42%	11.52%	27.67%	10.35%	34.70%	12.87%	29.73%
6	1,000	6.42%	28.99%	8.23%	24.77%	7.42%	30.63%	9.22%	27.11%
12	250	17.67%	29.27%	26.59%	34.45%	18.62%	29.71%	27.47%	35.52%
12	500	12.76%	23.38%	19.13%	30.34%	13.58%	23.87%	19.87%	31.45%
12	1,000	9.28%	19.15%	13.78%	27.04%	9.90%	19.74%	14.35%	28.26%

Length is the length of items; SS is the sample size, and RMSE is Root Mean Square Error without the degree of freedom adjustment.

It should be noted that this design is subject to a limitation. The performances of statistics, which are RMSE and %bias used in the original study, have different meanings when the true effects are different. For example, a 10%bias in a condition where the true effect is 0.731 can lead to a considerable number of studies establishing wrong predictions that view the internal consistency of a study as not acceptable because it could create a 95% distribution like [0.5, 0.9] and consequently yield wrong decisions based on these outputs. For instance, a researcher could consider a 0.6 measurement error of Alpha level in a study not acceptable. A 20%bias in the condition that the true effect is zero will not usually influence the decision-making process since it could create a 95% distribution like [0, 0.3]. In the scenario of a null distribution of internal consistency in the population, it does not matter whether the internal consistency is 0.1 or 0.2, as neither internal consistency score is acceptable in psychological studies.

We propose adding two new designs to the Monte Carlo simulation in psychological statistic testing to overcome this limitation: FNm and FPm.

### Simulation Study 2: estimating the modified false positive and modified false negative

First, it is essential to review the definition of FP and FN in the confusion matrix. As shown in Table 1, an accurate definition of FP is the percentage of results that erroneously indicate that a particular condition or attribute (e.g., correlation between variables in the same test) is present, whereas FN is the percentage of results that erroneously indicate that a particular condition or attribute is absent.

These two percentages can be used as criteria in binary outcomes. However, their usage with continuous results is problematic. FP and FN are originally designed for binary results (e.g., yes or no, acceptable or unacceptable). In computer science, the results tend to be clear and objective (i.e., an object is a dog or not a dog). However, this is not always the case in psychological science. The pre-determined cut-off used in psychology for binary conclusions is arbitrary. For instance, it is hard to justify why 0.69 is an unacceptable level of internal consistency while 0.70 is acceptable. This kind of binary thinking is often inappropriate in psychological research. It further implies that designing and measuring P (Internal consistency in simulation <0.70| Population internal consistency = 0.71) or P (Internal consistency in simulation >0.70| Population internal consistency = 0.69) becomes questionable since there is no substantive difference between an internal consistency of 0.69 and 0.70, in which P (X|Y) is a conditional probability, means the possibility of X in the condition of Y.

In addition, as discussed above, it is also meaningless to measure the percentage of internal consistency and report a weak relationship among variables when the internal consistency in the population follows a null distribution (i.e., P (Internal consistency in simulation > = 0.05| Population internal consistency = 0) and (Internal consistency in simulation <= 0.05| Population internal consistency = 0)). Thus, internal consistency close to 0.1 is not acceptable in psychological research.

Therefore, these FP and FN percentages have little practical meaning. However, there is a clear difference between the null distribution condition (e.g., Internal consistency = 0.0) and an acceptable level of relationship (e.g., Internal consistency = 0.7). Therefore, we propose two new metrics based on FP and FN, named FPm and FNm, and suggest a study similar to the original that additionally measures these metrics, in which FPm is the percentage that a statistic returns an acceptable level of statistics result when the statistic follows a null distribution in the population (6), and FNm is the percentage that a statistic returns a null result statistic when, in fact, the parameter is at an acceptable level in the population (7).


(6) FPm=P(Acceptable level ofastatistic in simulation|Null distribution of statistic).

(7) FNm=P(Null distribution of statistic in simulation|Acceptable level parameter in population).

The letter M in FPm and FNm stands for modification.

According to the percentage that should be measured, the FPm in this study is (8).


(8) $$P\;\left(\begin{array}{l} \mathrm{Internal consistency in simulation}>=0.7\;|\;\\ \mathrm{Population internal consistency parameter}=0.0 \end{array}\right).$$

The FNm in this study is (9).


(9) $$P\left(\begin{array}{l} \mathrm{Internal consistency in simulation}<=0.0\;|\;\\ \mathrm{Population internal consistency}=0.70 \end{array}\right)$$

RMSE is not applicable for this design. Yet, criteria are needed for the purpose of this new design. Hence, we propose two criteria:

Ideally, FPm and FNm should be close to 0% across all conditions. Therefore, for comparison between statistics, the fewer the number of conditions having a number larger than zero, the better the statistic.

In addition, suppose that FPm or FNm is larger than 5% in a certain condition. We suggest that the statistic should be deemed questionable in this condition and not used. This suggestion is based on the standard tolerable level of binary decision error. For instance, if a statistic shows an FPm of 0.3 when the sample size is 200, we would propose that this statistic is unreliable in this sample size condition because an acceptable level of relationship can be reported by this statistic even if the statistic in the population follows a null distribution. However, this statistic could be reliable with a sample size of 1,000, depending on TP, TN, FPm, and FNm values following this rationale. As a result, we suggest that extreme conditions in psychological research should be included in the simulation study to provide comprehensive results.

### Design

At first, for both FPm and FNm, the following conditions were included in our study as the original study did: the four internal consistency indices and questionnaire lengths of 6 and 12 items. We included 250, 500, and 1,000 for sample size. In addition, small sample sizes of 20, 25, 30, 35, 40, 45, and 50 are included in this study to test whether there is any condition in psychological studies that these biases will influence TN results.

In the evaluation of FPm, the datasets were simulated with the same population [i.e., N (0.1)] as in Study 1 to create the null distribution of statistics. In the evaluation of FNm, the datasets were simulated with the same method implemented in the original study. This makes the overall conditions 7*2 = 14. Both tau-equivalent and congeneric models are included. The population covariance matrixes are displayed in the code. All four statistics in the original study are included with questionnaire lengths of 6 and 12. Consequently, this makes the overall conditions 14 in FPm and 28 in FNm.

## Results

The simulation results of FPm are displayed in Table 3, while the results of FNm are displayed in Table 4. As can be seen in Table 3, *based* on the criteria we proposed, (1) Alpha performs best when there is a null distribution in the internal consistency, and (2) the acceptable level of results of Omega, GLB, and GLBa is questionable when the sample size is less than 30 to 40, depending on the questionnaire length. As can be seen in Table 4, based on the criteria we proposed, all internal consistency indices showed good FNm. This suggests that, under the conditions of our study using the four indices, a result close to zero is highly unlikely to originate from a population with an acceptable level of internal consistency.

**Table 3:** Estimation of false positive modified in Study 2.

Length	SS	Alpha	Omega	GLB	GLBa
6	20	0.19%	**49.93%**	**11.07%**	**8.48%**
6	30	0.00%	**16.55%**	1.73%	1.85%
6	40	0.00%	**5.43%**	0.22%	0.36%
6	50	0.00%	1.63%	0.07%	0.16%
6	250	0.00%	0.00%	0.00%	0.00%
6	500	0.00%	0.00%	0.00%	0.00%
6	1,000	0.00%	0.00%	0.00%	0.00%
12	20	0.89%	**40.98%**	**85.25%**	**35.37%**
12	30	0.00%	**5.03%**	**42.15%**	**11.59%**
12	40	0.00%	0.32%	**14.03%**	3.36%
12	50	0.00%	0.02%	3.70%	0.98%
12	250	0.00%	0.00%	0.00%	0.00%
12	500	0.00%	0.00%	0.00%	0.00%
12	1,000	0.00%	0.00%	0.00%	0.00%

Length is the length of items; SS is the sample size. The percentage values are acceptable (i.e., adequate reliability) when the dataset follows a null distribution (i.e., zero reliability) in the population. Percentages in bold are the Percentages above 5%, which suggests the result of a specific statistic is questionable in this condition.

**Table 4:** Estimation of false negative modified in Study 2.

QL	SS	Condition	Alpha	Omega	GLB	GLBa
6	20	TE	0.28%	0.00%	0.00%	0.00%
6	20	CG	0.34%	0.00%	0.00%	0.00%
12	20	TE	0.01%	0.00%	0.00%	0.00%
12	20	CG	0.01%	0.00%	0.00%	0.00%
6	30	TE	0.09%	0.00%	0.00%	0.00%
6	30	CG	0.15%	0.00%	0.00%	0.00%
12	30	TE	0.03%	0.00%	0.00%	0.00%
12	30	CG	0.01%	0.00%	0.00%	0.00%
6	40	TE	0.03%	0.00%	0.00%	0.00%
6	40	CG	0.03%	0.00%	0.00%	0.00%
12	40	TE	0.01%	0.00%	0.00%	0.00%
12	40	CG	0.00%	0.00%	0.00%	0.00%
6	50	TE	0.00%	0.00%	0.00%	0.00%
6	50	CG	0.03%	0.00%	0.00%	0.00%
12	50	TE	0.02%	0.00%	0.00%	0.00%
12	50	CG	0.02%	0.00%	0.00%	0.00%
6	250	TE	0.00%	0.00%	0.00%	0.00%
6	250	CG	0.01%	0.00%	0.00%	0.00%
12	250	TE	0.00%	0.00%	0.00%	0.00%
12	250	CG	0.00%	0.00%	0.00%	0.00%
6	500	TE	0.00%	0.00%	0.00%	0.00%
6	500	CG	0.01%	0.00%	0.00%	0.00%
12	500	TE	0.00%	0.00%	0.00%	0.00%
12	500	CG	0.00%	0.00%	0.00%	0.00%
6	1,000	TE	0.00%	0.00%	0.00%	0.00%
6	1,000	CG	0.06%	0.00%	0.00%	0.00%
12	1,000	TE	0.00%	0.00%	0.00%	0.00%
12	1,000	CG	0.00%	0.00%	0.00%	0.00%

QL is the length of items; SS is the sample size. TE is tau-equivalent model. CG is Congeneric model. Percentage values are failures that suggest statistics report that there is no internal consistency when in fact, there is an acceptable internal consistency in the population.

## Discussion

Our study, alongside the original study by Trizano-Hermosilla and Alvarado (2016), presents a new Monte Carlo simulation design within the confusion matrix paradigm. We have proposed new conditions, guided by the perspective of the confusion matrix, that should be included in the evaluation of statistical simulation studies. Firstly, we will discuss the findings of internal consistency indices. Secondly, we will provide a summary of how to apply this novel confusion matrix design to simulation studies in statistics comparison. Thirdly, we will engage in a general discussion.

### Issues of internal consistency indices

This study is not primarily focused on which kind of internal consistency indices should be used in psychological research. Therefore, the study has replicated the design of the original study (i.e., sample size and questionnaire length) when applicable to provide an example of how to apply this confusion matrix design. This does not imply that we see no space for improvement in the conditions included in the study. For instance, Likert scale variables should be included in the simulation as internal consistency indexes are usually applied to the Likert scale variables in psychological research (Croasmun and Ostrom, 2011). However, we have found additional empirical evidence that should be used as a reference for the performance of these statistics. Through this additional evidence, we have found that Omega and GLB indices do not perform well enough for small sample sizes under some conditions. Yet, our results do not imply that Alpha should necessarily be preferred over Omega. We admit that Alpha has shortcomings as an index for measuring internal consistency, which is boosted by the length of the questionnaire or prerequisites that are violated, as described in previous studies (McNeish, 2018; Hayes and Counts, 2020).

However, we have found that under some conditions (e.g., sample size = 20, 30, or 40), Omega.total and GLB are boosted and thus become unreliable. Specifically, it is difficult to distinguish a population with random numbers from a population that has high internal consistency. Therefore, in these conditions (i.e., sample size <40), we recommend that Omega.total and GLB be avoided in estimating the internal consistency, no matter what kind of performance Omega.total has when there is an acceptable level of the parameters in a given population. These suggestions are based on the results of this simulation study, which are limited by the study’s design.

We simulated a null distribution for internal consistency, specifically using a normal distribution generated randomly for each item. This implies that all effects in the dataset are essentially noise. To our understanding, the reason why the Omega statistic tends to be inflated in small sample sizes is due to its value range being restricted to [0,1]. Consequently, any noise in the dataset disproportionately affects Omega positively. As suggested by Okada (2017), the zero-winsorized method can create positive biases. Especially in conditions of small sample sizes, such biases can lead to inflated results, sometimes even exceeding the established cut-off (i.e., 0.7).

Moreover, related Omega indices, such as Hierarchical Omega, should also be tested when researchers aim to measure the reliability of the general factor only. All these indices with these conditions should be tested through the TP and TN conditions, corresponding to FPm and FNm. Most importantly, all these conditions should be tested simultaneously in a simulation study to provide empirical evidence for applied researchers. Suppose the proposed design had been applied in the original study. A more conservative recommendation of Omega with a discussion of Omega’s limitations will be provided in the original study and studies influenced by the original study (Watkins, 2017; McNeish, 2018; Cortina et al., 2020).

### Practical recommendations and steps when implementing a confusion matrix design through Monte Carlo simulation

Step 1: Both conditions in which there is a certain relationship between variables and the condition in which the expected association is deemed as absent should be included in the simulation design (i.e., the null distribution of statistics), together with other relevant criteria such as sample size, distribution, and alike. These two kinds of conditions ought to be included as TP and TN, respectively.

In simulating the null distribution of statistics, we advocate for consistently employing the method outlined in the APA guidelines (Fan, 2012). This approach ensures that the simulation design accurately represents a population with a null statistic distribution and assesses its impact on the observed sample statistics. Our findings confirm that it is possible to reconstruct an estimation by a normally distributed dataset in the absence of internal consistency across four reliability statistics, which have theoretical and practical implications that are related to the definition and calculation of what is considered to be a large sample. For instance, as described in Study 1, we calculated all four statistics (i.e., Omega, Alpha, GLB, and GLBa) with a large sample of 100,000 and a standard normal distribution, ensuring the inclusion of a null distribution of statistics since all the statistics are close to zero in such an extensive sample.

Meanwhile, it’s important to acknowledge that there are various types of null distributions for a statistic. Although our simulation study only includes normal distributions, we encourage researchers to explore a broader range of nonnormal distributions. This expansion is crucial to estimating the robustness of statistics under a variety of True Negative (TN) conditions. When doing so, researchers should employ the checking method we mentioned earlier to ensure that the design excludes any relationship specific to the statistic being tested.

Step 2: Suppose there is a commonly used cut-off or an acceptable level of a statistic with a continuous result. FPm (5) and FNm (6) should be measured in various conditions, such as those conditions commonly occurring in practice.

We have already described the difficulty of practicing FN and FP directly in statistics used in psychology. Yet, we also admit that FNm is necessary but not sufficient to estimate FN. Analogically speaking, using FNm to replace FN and using FPm to replace FP would be like trying to measure whether an unknown number X is bigger than 1 to solve the question of whether X > 2. If X ≤ 1, then X is definitely less than 2. However, if X > 1, it does not necessarily mean X is greater than 2.

The confusion matrix design also works in this way. Suppose a statistic can report a result above the cut-off or an acceptable level of a relationship between variables measured by this statistic when there is a null distribution of the statistics in this condition. In this case, it is also highly likely that the statistic will report a result above this cut-off when the population parameter is lower than the acceptable level. As a result, the statistics in this condition are not reliable. To estimate the possibility of this situation, we conducted another simulation study that used internal consistency levels of 0.3 and 0.5 as the true parameters of the population. The result is in Table 5. According to our findings, the Omega is also boosted in the conditions tested as questionable by FPm. Therefore, FPm scores above 5% are reliable enough to ascertain when a statistic should be considered questionable. Some researchers might argue that this part of the simulation may also be included in our proposed confusion matrix design. Yet, for some statistics, it is not easy to find a present but not acceptable level of the statistic.

**Table 5:** Estimation of false positive method with unacceptable internal consistency level.

QL	SS	Condition	IL	Alpha	Omega	GLB	GLBa
6	20	TE	0.3	0.75%	**56.45%**	**35.83%**	**34.83%**
6	25	TE	0.3	0.20%	**39.08%**	**23.99%**	**25.93%**
6	30	TE	0.3	0.12%	**27.14%**	**16.02%**	**19.77%**
6	35	TE	0.3	0.02%	**18.08%**	**10.25%**	**14.81%**
6	40	TE	0.3	0.01%	**12.68%**	**6.28%**	**10.77%**
6	45	TE	0.3	0.02%	**8.89%**	4.12%	**8.35%**
6	50	TE	0.3	0.00%	**6.63%**	2.60%	**6.68%**
6	250	TE	0.3	0.00%	0.00%	0.00%	0.00%
6	500	TE	0.3	0.00%	0.00%	0.00%	0.00%
6	1,000	TE	0.3	0.00%	0.00%	0.00%	0.00%
12	20	CG	0.5	6.82%	**60.38%**	**99.63%**	**92.11%**
12	25	CG	0.5	3.65%	**40.39%**	**98.48%**	**88.77%**
12	30	CG	0.5	2.07%	**28.16%**	**96.93%**	**85.42%**
12	35	CG	0.5	1.21%	**21.08%**	**95.50%**	**82.38%**
12	40	CG	0.5	1.07%	**16.04%**	**93.19%**	**79.36%**
12	45	CG	0.5	0.60%	**12.09%**	**90.52%**	**76.02%**
12	50	CG	0.5	0.47%	**9.49%**	**87.67%**	**73.44%**
12	250	CG	0.5	0.00%	0.00%	2.18%	**18.57%**
12	500	CG	0.5	0.00%	0.00%	0.00%	7.78%
12	1,000	CG	0.5	0.00%	0.00%	0.00%	3.43%

QL is the length of the questionnaire or the item number in a questionnaire; SS is the sample size. TE is tau-equivalent model. CG is Congeneric model. IL is the population internal consistency parameter of Alpha. Percentage values are failures that suggest that a statistic report that internal consistency is above the cut-off when in fact, there is an internal consistency parameter that is considerably away from this cut-off.

Furthermore, our research identified two key relationships between True Negative (TN) and FPm. If a statistic shows poor performance in the TN condition, it is likely to also fare poorly in the FPm condition. This observation aligns with the rationale we discussed earlier. Additionally, we found that a positive bias in TN is correlated with an increased likelihood of simulation study results meeting the acceptable cut-off. Using the original study as an empirical example of True Positive (TP), we can reasonably infer that all four indices demonstrate robust performance in FNm. Thus, for statistics without a pre-established cut-off, we recommend using TN and TP as predictive references. A large absolute value in percentage bias and RMSE suggests that the statistical output is likely derived from population samples.

### Several research scenarios

We have demonstrated a comprehensive example of applying this enhanced confusion matrix design in evaluating internal consistency indices. To further clarify, we propose that this design is versatile and can be applied to a broader range of tasks. Before delving into a general discussion, we will present three concise examples illustrating how the confusion matrix design can be implemented in other published simulation studies. In the first two studies, only TN conditions can be applied, as these studies do not have a common cut-off for their respective statistics (i.e., correlation coefficients and mediation correlation coefficients). However, for the third study, we will apply the full confusion matrix design, as it involves a cut-off for Root Mean Square Error of Approximation (RMSEA) in Structural Equation Modeling (SEM).

Ventura-León et al. (2023) executed a Monte Carlo simulation study focusing on correlation coefficients commonly used in psychology research. They examined various population correlation conditions, such as 0.12, 0.20, 0.31, and 0.50, under nonnormal distributions and distributions with outliers. Their findings indicated that the Winzorized Pearson correlation coefficient (Wilcox, 2011) performed the best within the simulated conditions they included. Based on the design of our study, we suggest that Ventura-León et al. (2023) should also consider including conditions with a null distribution of the statistics, specifically where the population correlation is zero that can be used as TN. The absence of TN in their study leaves a gap in empirical evidence regarding the performance of correlation coefficients under this condition. This omission poses a risk, as certain correlation coefficients may exhibit poor performance at the zero point, like the Eta square effect size (Okada, 2013) and the Omega statistics in our simulation.

Sim et al. (2022) conducted a Monte Carlo simulation study to estimate the necessary sample size for detecting mediation effects in various models. Their study included partial and full mediation conditions, providing the minimum sample size required to detect these effects. However, their design overlooked the inclusion of null distribution of mediation effects conditions, which are crucial for assessing the sample size needed to maintain a reasonable Type-I error level. This omission can bring significant problems. For instance, suppose a sample size requirement of 200 is found under some null distribution conditions to ensure the correct result is found in most replications. Then, the conclusions of Sim et al. (2022) might be called into question. They concluded that a sample size of 90 is sufficient to detect a mediation effect when the factor loading is 0.7 with a large effect size. Yet, this sample size level may not avoid the detection of a mediation effect in a population where no such effect exists. Including conditions with no mediation effect, as TN proposed in our study, is essential to test and validate the sample size requirements thoroughly.

In the case of the studies by Ventura-León et al. (2023) and Sim et al. (2022), the simulation conditions of FPm and FNm are not applicable, as these studies lack defined criteria for determining satisfactory levels of mediation effect or correlation coefficients. Next, we will examine another study by Gao et al. (2020), which focuses on the RMSEA in SEM. Our discussion will first detail the design of Gao, Shi, and Maydeu-Olivares’s study, followed by its shortcomings. We will then explore how the methodology of our study can be applied to theirs to address these limitations.

Gao et al. (2020) used a Monte Carlo simulation study to examine the robustness of several RMSEA measurements. Their studies have included several robust RMSEA measurement methods and conditions with normal and nonnormal distributions. They found that RMSEA with mean and variance corrections is the most robust as it performs best across all conditions.

From our perspective, the study conducted by Gao et al. (2020) has shortcomings. One significant limitation is their failure to test the statistics under a null distribution condition, such as a simulated distribution in which items bear no relationship to the model. This omission means that they have not provided empirical evidence about the performance of these statistics in such a null condition. Therefore, it is essential to include TN conditions in their analysis. Additionally, they should test whether any RMSEA correction methods can yield results considered a good fit under null distribution conditions. This FPm design could be assessed using a cut-off of 0.08, as Fabrigar (1999) suggested, across various conditions. If certain conditions reveal a good fit using an RMSEA correction method, then the performance of these statistics under these specific conditions becomes questionable. A similar approach could be applied to assess FNm.

## General discussion

This study introduces a novel simulation design based on a confusion matrix framework. As we propose, this innovative design is particularly suited for use in simulation studies that focus on comparing the performance of statistical methods under various conditions. To demonstrate its applicability, we have presented three potential scenarios and a detailed example illustrating the implementation of this design.

It is somewhat surprising that researchers might overlook the fact that studies like the original one can only yield empirical evidence when the attribute under investigation reaches an acceptable level. Consider a hypothetical scenario where all populations in psychological research exhibit an acceptable level of a particular statistical parameter. In such a case, regardless of whether the original study violated any assumptions, there would be no necessity to develop statistics to verify the existence of an effect. Furthermore, it’s important to reiterate that APA guidelines advise researchers to include a null effect in any simulation of effect, specifically the absence of a mean difference between two groups (Fan, 2012). However, the rationale provided by the APA primarily aims to prevent Type-I errors, potentially leading researchers to mistakenly believe that the null distribution of statistics is only relevant for inferential statistics closely related to NHST. Our research findings suggest otherwise; different statistics may perform variably under different conditions. Identifying the most suitable statistic for these conditions requires including these conditions with an evaluation of the commonly used criteria.

## Data availability statement

The original contributions presented in the study are included in the article/Supplementary material, further inquiries can be directed to the corresponding author.

## Author contributions

YC: Data curation, Writing – original draft. PP-D: Writing – review & editing. KP: Writing – review & editing. JL: Writing – review & editing.

## Appendix

The R code that generated all the data and simulation results in this study is available in a separate file that is attached to the current submission to the journal Frontiers in Psychology. It is also available through the URL: https://liqas.org/code-under-review/. Researchers are encouraged to simulate and replicate the results for future research. This study was not preregistered.
